# Synthesis, in silico molecular docking analysis, pharmacokinetic properties and evaluation of antibacterial and antioxidant activities of fluoroquinolines

**DOI:** 10.1186/s13065-022-00795-0

**Published:** 2022-01-13

**Authors:** Mona Fekadu, Digafie Zeleke, Bayan Abdi, Anuradha Guttula, Rajalakshmanan Eswaramoorthy, Yadessa Melaku

**Affiliations:** 1 Departments of Applied Chemistry, Adama Science and Technology University, P.O.Box 1888, Adama, Ethiopia; 2 Department of Biomaterials, Saveetha Dental College and Hospitals, Saveetha Institute of Meidcal and Technical Sciences (SIMATS), Saveetha University, Chennai, 600 077 India

**Keywords:** Fluoroquinolines, Antibacterial, Antioxidant, Anticancer, Molecular docking

## Abstract

**Background:**

Quinolines have demonstrated various biological activities such as antimalarial, antibacterial and anticancer. Hence, compounds with such scaffold have been used as lead in drug development. This project is, therefore, aimed to synthesis and evaluates some biological activities of quinoline analogs.

**Methods:**

2-Chloro-7-fluoroquinoline-3-carbaldehydes were synthesized by the application of Vilsmeier–Haack reaction. The chlorine in the fluoroquinoline-3-carbaldehyde was replaced with various nucleophiles. The aldehyde functional group was also converted to carboxylic acid and imine groups using oxidizing agent and various amines, respectively. The structures of the compounds synthesized were characterized by spectroscopic methods. Disc diffusion and DPPH assays were used to evaluate the antibacterial and antioxidant activities, respectively. The in silico molecular docking analysis of the synthesized compounds were done using AutoDock Vina against *E. coli* DNA Gyrase B and human topoisomerase IIα. The drug likeness properties were assessed using SwissADME and PreADMET.

**Results:**

Nine novel quinoline derivatives were synthesized in good yields. The in vitro antibacterial activity of the synthesized compounds was beyond 9.3 mm inhibition zone (IZ). Compounds **4**, **5**, **6**, **7**, **8**, **10**,** 15**, and **16** exhibited activity against *E. coli*, *P. aeruginosa*, *S. aureus* and *S. pyogenes* with IZ ranging from 7.3 ± 0.67 to 15.3 ± 0.33 mm at 200 μg/mL. Compound **9** displayed IZ against three of the bacterial strains except *S. aureus*. The IC_50_ for the radical scavenging activity of the synthesized compounds were from 5.31 to 16.71 μg/mL. The binding affinities of the synthesized compounds were from − 6.1 to − 7.2 kcal/mol against *E. coli* DNA gyrase B and − 6.8 to − 7.4 kcal/mol against human topoisomerase IIα. All of the synthesized compounds obeyed Lipinski’s rule of five without violation.

**Conclusion:**

Compounds **4**, **5**, **6**, **7**, **8**, **10**, **15**, and **16** displayed activity against Gram positive and Gram negative bacterial strains indicating that these compounds might be used as broad spectrum bactericidal activity. Compound **8** (13.6 ± 0.22 mm) showed better IZ against *P. aeruginosa* compared with ciprofloxacin (10.0 ± 0.45 mm) demonstrating the potential of this compound as antibacterial agent against this strain. Compounds **5**, **6**, **7**, **8**, **9** and **10** showed comparable binding affinities in their in silico molecular docking analysis against *E. coli* DNA gyrase B. All of the synthesized compounds also obeyed Lipinski’s rule of five without violation which suggests these compounds as antibacterial agents for further study. Compounds **7** and **8** were proved to be a very potent radical scavenger with IC_50_ values of 5.31 and 5.41 μg/mL, respectively. Compound **5**, **6**,** 8**,** 10** and **16** had comparable binding affinity against human topoisomerase IIα suggesting these compounds as a possible candidate for anticancer drugs.

**Supplementary Information:**

The online version contains supplementary material available at 10.1186/s13065-022-00795-0.

## Introduction

Microbial infections remain a serious health threat throughout the world even in the modern era [1]. Microbial pathogens cause more than 400 million deaths annually across the world [2]. So far, several modern antimicrobial agents have been developed and used for the treatment of various infections and have saved millions of lives [3]. The beginning of the modern “antibiotic era” pioneered by Paul Ehrlich and Alexander Fleming was meant to synthesize chemical compounds which could destroy only the parasite harbored within the organism without affecting the host significantly. This breakthrough led to the development of a large-scale and systematic screening program in 1904 to find a drug against syphilis, a disease that was endemic and almost incurable at that time [3]. Nowadays, there are a large number of natural, semisynthetic and synthetic antimicrobial agents [4]. Drugs based on quinoline scaffold were among the fully synthetic antimicrobial agents.

Quinoline is a heterocyclic aromatic compound that has been used to develop various antimicrobial agent [5]. Quinoline scaffold has been used to develop antimalarial [6], antibacterial [7] and anticancer [8] drugs. However, despite the development of several types of antimicrobial agents, the fight against pathogenic microbes is not complete yet. Antimicrobial resistance (AMR) has been one of the major public health concerns of the twenty-first century [9]. It refers to resistance developed by pathogenic microorganisms to antimicrobial drugs which makes drugs ineffective [10]. AMR partly caused by prolonged use of antimicrobials and antibiotics for therapeutic use. The death toll caused by AMR is estimated to be well over 700,000 each year globally [11].

Thus, developing new antimicrobial agents and increasing the effectiveness of the existing one by structural modification can play profound role to combat multidrug resistance diseases. In this regard, quinoline scaffold has been used to design new prototypes of drug-candidates with different biological activities [12]. Thus, many quinoline analogs have been developed and their structural modifications have helped in improving biological activities. It was in 1960s that the first generation quinolones from quinine showed antibacterial activities against Gram negative bacteria [13]. Structural modification of the first generation quinolones by the addition of a fluorine atom at position 6 and piperazine to the C-7 position increased their bioactivity significantly and provided the second generation fluoroquinolones [14]. Further study of their structural activity and modification of their structures yielded the third and fourth generation fluoroquinolones which displayed broad spectrum activities than their former analogs [7, 15]. At the same time, several 2-chloroquinoline-3-carbaldehyde derivatives were synthesized wherein their biological activities were determined. But, none of them showed strong biological activities to be patented for antimicrobial agents [16, 17]. Therefore, anticipating the same trend in quinoline-3-carbaldehyde derivatives with fluoroquinolone, several novel fluoroquinoline derivatives were synthesized and their in vitro antibacterial and antioxidant activities were evaluated. Moreover, in silico molecular docking analysis, drug-likeness and toxicity prediction were also conducted.

## Methods

### General

Analytical grade chemicals were purchased from Lova Chemie PVT LTD and used without additional purification. Melting points were determined by Thiele tube expressed in °C. The progress of the reactions were monitored with TLC and spots were visualized using UV light at 254 nm. Silica gel (60–120 mesh, Merck) has been used for column chromatography. The synthesized compounds were characterized on the basis of physical and spectral analysis. The UV–Vis spectra of synthesized compounds were recorded on Double-beam UV–Vis spectrophotometer using DCM and MeOH as blank solvents and λ_max_ values were expressed by nm. The ^1^H and ^13^C NMR spectra of the synthesized compounds were recorded on Bruker avance 400 MHz NMR spectrophotometer using chloroform-*d* or methanol-*d*_*4*_ as the solvent and the values are expressed in *δ* ppm.

### Experimental procedures

The synthesis of the compounds synthesized in the present work was in accordance with the protocol reported by Zeleke et al. [18] with slight modifications.

#### Synthesis of *N-*(4-flouro phenyl) acetamide (3)

4-Fluoro aniline (15 mL), acetic anhydride (22 mL), zinc powder (0.1 g, 0.0016 mmol) and glacial acetic acid (23 mL) were added in 250 mL round bottom flask. The mixture was boiled by refluxing using water condenser for 2 h. Then, it was cooled to room temperature and poured into 200 mL of crushed ice water. The solid product, *N-*(4-flouro phenyl) acetamide was collected by suction filtration. White crystal; yield 19.69 g (81.2%); mp 154–156 °C.

#### Synthesis of 2-chloro-6-fluoro quinoline-3-carbaldehyde (4)

*N,N*-dimethylformamide (29.9 mL) was added to a 100 mL round-bottom flask guarded with drying tube; it was cooled to 0 °C using ice bath. Then, phosphorus oxychloride (84.3 mL) was added dropwise to it from dropping funnel guarded by drying tube while being stirred by magnetic stirrer. This addition was done for 30 min. Then *N*-(4-flouro phenyl) acetamide (19.69 g, 0.00012 mmol) was added to it after 5 min. The dropper funnel was replaced by air condenser with guarding tube at its end and the mixture was heated for 22 h on oil bath at 85–90 °C. Then it was cooled to room temperature, poured into a beaker containing 400 mL crushed ice water, and stirred for 20 min. The yellow solid product was collected by suction filtration and washed with 100 mL cold water. The crude yield was 16.4 g (60.8%) and was recrystallized from ethyl acetate. yellow crystal; mp 160^_^162 °C; yield 15.5 g (57.5%); *R*_f_ 0.72 (*n*-hexane:EtOAc, 4:1); ^1^H NMR (400 MHz, CDCl_3_): *δ* 9.36 (1H, *s*, H-9), 7.54 (1H*, s*, H-4), 6.85 (1H, *dd*, *J* = 12.8 Hz and 3.2 Hz, H-7), 6.47 (1H, *d*, *J* = 12 Hz, H-8), 6.32 (1H, *d*, *J* = 8.4 Hz, H-5); ^13^C NMR (101 MHz, CDCl_3_): *δ* 189.1 (C-9), 162.2 (C-6), 148.6 (C-2), 143.9 (C-8a), 139.7 (C-4), 130.77 (C-8), 130.0(C-4a), 127.6 (C-3), 123.8 (C-7), 111.2 (C-5).

#### Synthesis of 6-fluoro-2-methoxy quinoline-3-carbaldehyde (5)

The methanol (10 mL), *N,N-*dimethylformamide (13 mL), 2-chloro-6-flouro quinoline-3-carbaldehyde (0.5 g, 0.00024 mmol) and potassium carbonate (0.57 g, 0.00041 mmol) were added to A 100 mL two-neck round bottom flask. The mixture was refluxed in water bath for 4 h with the progress monitored with TLC. After completion of the reaction, methanol was removed by distillation, cooled to room temperature and then added to 100 mL ice-cold water. The solid product was collected by fractional distillation and washed with excess ice-cold water. Yellow powder; yield 75.2%; mp 158–160 °C; *R*_f_ 0.78 (*n*-hexane:EtOAc,4:1); ^1^H NMR (400 MHz, CDCl_3_): *δ* 10.46 (1H, *s*, H-9), 8.50 (s, 1H, H-4), 7.83 (1H, *d*, *J* = 9.2 Hz, H-8), 7.51 (2H, *m*, H-5 and H-7), 4.17 (3H, *s*, OMe); ^13^CNMR (101 MHz, CDCl_3_): *δ* 189.1 (C-9), 160.7 (C-2), 158.0 (C-6), 146.0 (C-8a), 139.0 (C-4), 129.4 (C-8), 124.7 (C-4a), 122.2 (C-7), 120.7 (C-3), 112.7 (C-5), 54.0 (C-11).

#### Synthesis of 2-ethoxy-6-flouro- quinoline-3-carbaldehyde (6)

A 2-chloro-6-flouro quinoline-3-carbaldehyde (0.5 g, 0.00026 mmol), potassium carbonate (0.6 g, 0.00043 mmol), ethanol (10 mL) and *N,N*-dimethylformamide (10 mL) were added to 100 mL two-neck round-bottom flask and the necks were snug with water condenser and stopper. The mixture was refluxed for 4 h whereby the progress was monitored with TLC. At the end, the ethanol was removed by distillation, and the remaining cooled mixture was poured into 100 mL crushed ice water. The solid mass was collected by suction filtration. Yield 72.3%; white powder; mp 184–186 °C, *R*_*f*_ 0.64 (*n-*hexane:EtOAc, 4:1); ^1^H NMR (400 MHz, CDCl_3_): *δ* 10.51 (1H, *s*, H-9), 8.52 (1H, *s*, H-4), 7.84 (1H, *dd, J* = 8.4 Hz and 1.6 Hz, H-7), 7.49 (2H, *m*, H-5 and H-8), 4.64 (2H, *q*, *J* = 7.1 Hz, H-10), 1.52 (3H, *t*, *J* = 7.1 Hz, H-11); ^13^C NMR (101 MHz, CDCl_3_): *δ* 189.3 (C-9), 160.6 (C-2), 158.0 (C-6), 145.9 (C-8a), 138.7(C-4), 129.3 (C-8), 124.6 (C-4a), 122.1 (C-7), 120.4 (C-3), 112.7 (C-5), 62.7 (C-10), 14.3 (C-11).

#### Synthesis of 6-fluoro-2-thiocyanatoquinoline-3-carbaldehyde (7)

Potassium thiocyanate (0.24 g, 0.00025 mmol), 2-chloro-8-methyl quinoline-3-carbaldehyde (0.4 g, 0.00020 mmol),and potassium carbonate (0.65 g, 0.00047 mmol) were added to 100 mL two-neck round-bottom flask containing *N,N*-dimethylformamide (20 mL). One of its necks was connected to a condenser, and the other was closed with glass stopper and then refluxed for 5 h and progress was checked by TLC. It was cooled to room temperature and poured into 50 mL crushed ice water. The solid product was collected with suction filtration and washed with 10 mL cold water. Yield 67%; Gray powder; mp 156–158 °C; *R*_f_ 0.49 (DCM:Methanol, 99:1); ^1^H NMR (400 MHz, DMSO-d6): *δ* 10.24 (1H, *s*, H-9), 8.48 (1H, *s*, H-4), 7.81 (1H, *dd*, *J* = 9.2 Hz and 2.4 Hz, H-7), 7.57 (1H, *m*, H-8), 7.37 (1H, *m*, H-8); ^13^C NMR (101 MHz, DMSO-d6): *δ* 190.2 (C-9), 161.6 (C-2), 158.7 (C-6), 156.2 (C-8a), 142.0 (C-4), 138.4 (C-4a), 126.9 (C-3), 122.5 (C-8), 119.1 (C-10), 117.8 (C-7), 115.6 (C-5).

#### Synthesis of 2-chloro-6-fluoroquinoline-3-carboxylic acid (8)

Sodium hydroxide solution (1 mL, 10%) was added to a suspensions of 2-chloro-6-fluoroquinoline-3-carbaldehyde (0.5 g, 0.00026 mmol) in water (20 mL). Then, saturated solution of potassium permanganate was added dropwise until a definite purple color remained after quivering the solution. The mixture was acidified with 10% of sulfuric acid and oxalic acid was added to destroy the excess permanganate solution. The carboxylic acid precipitate was collected by suction filtration. 63.1% yield, Yellow powder, mp 200–202 °C; *R*_f_ 0.67 (*n-*hexane:EtOAc, 4:1); ^1^H NMR (400 MHz, CDCl_3_): *δ* 11.00 (1H, *s*, H-9), 9.13 (1H, *s*, H-4), 8.51 (1H, *d*, *J* = 11.3 Hz, H-8), 8.06 (1H, *d*, *J* = 3 Hz, H-5), 7.72 (1H, *dd*, *J* = 8.2 Hz, H-7); ^13^C NMR (101 MHz, CDCl_3_): *δ* 179.9 (C-9) 153.3 (C-6), 140.5 (C-2), 137.7 (C-8a), 130.5 (C-4), 122.2 (C-8), 118.2 (C-3 and C-4a), 114.8 (C-7), 103.7 (C-5)

#### Synthesis of 2-((2-hydroxyethyl) amino)-3-(-2-(ethylideneamino) ethanol quinoline (9)

2-Chloroquinoline-3-carbaldehyde (0.5 g, 0.00026 mmol) was added to ethanolamine (8 mL) in 100 mL round bottom flask and heated in oil bath at 100 °C for 2 h after cooling to ambient temperature, it was poured into 100 mL crushed ice water the precipitate was collected by suction filtration and allowed to dry in air. Yield 63.6%; yellow powder; mp 150–152 °C; *R*_f_ 0.57 (DCM:methanol, 99:1); ^1^H NMR (400 MHz, DMSO-d6): *δ* 9.49 (1H*, s*, H-9), 8.48 (1H, s, H-4), 8.18 (1H, *d*, *J* = 2.3 Hz, H-5), 7.52 (3H, *m,* H-7, H-8 and NH), 4.88 (2H, *m*, H-11), 3.5 (6H, *bro. s*, H-12, H-15 and H-16); ^13^C NMR (101 MHz, DMSO-d6): *δ* 163.7 (C-9), 158.3 (C-2), 155.0 (C-6), 145.3 (C-8a), 142.2 (C-4), 127.8 (C-8), 122.2 (C-4a), 122.1 (C-3), 120.6 (C-7), 111.9 (C-5), 63.6 (C-11), 61.2 (C-16), 60.3 (C-12), 43.4 (C-15).

#### Synthesis of 6-fluoro-*N*-phenyl-3-((phenylimino) methyl) quinolin-2-amine (10)

2-Chloroquinoline-3-carbaldehyde (0.5 g, 0.00026 mmol), aniline (8 mL) and acetic acid (9 mL) refluxed for 25 min. After cooled to ambient temperature, it was poured into 100 mL crushed ice water and precipitate was collected by suction filtration and allowed to dry in air. Yield 1.09 g (70%); Yellow powder; mp 162^_^164 °C; *R*_f_ 0.5 (*n-*hexane:EtOAc, 4:1); ^1^H NMR (400 MHz, DMSO-d6): *δ* 9.93 (1H, *s*, H-4), 7.59 (2H*, bro s*, H -8 and H-9), 7.57 (2H, *m*, H-5 and H-7), 7.00–7.29 (10H, t, phenyl), 7.26 (1H, *dd*, *J* = 7.6 Hz, H-7); ^13^C NMR (101 MHz, DMSO-d6): *δ* 161.9 (C-9), 157.3, 145.9, 153.9, 153.8, 153.7, 142.9, 140.8, 140.7, 124.9, 124.8, 120.7, 120.6, 118.4, 118.3, 116.4, 109.6, 109.4

#### Synthesis of 2-methoxyquinoline-3-carboxylic acid (15)

Sodium hydroxide solution (1 mL, 10%) was added to a suspension of 2-chloroquinoline-3-carbaldehyde (0.5 g, 0.00028 mmol) in water (20 mL). Then, saturated solution of potassium permanganate in water was added dropwise until a definite purple color remained after shaking the solution. The mixture was acidified with 10% of sulfuric acid to which oxalic acid was added to get rid off the excess permanganate solution. The carboxylic acid precipitate was collected by suction filtration. 60.1% yield, white powder, mp 205–207 °C; *R*_f_ 0.71 (*n-*hexane:EtOAc, 4:1) and then a 100 mL two-neck round-bottom flask was charged with methanol (10 mL) *N,N-*dimethylformamide (13 mL), 2-chloroquinoline-3-carboxylic acid (0.5 g) and potassium carbonate (0.57 g); the mixture was refluxed using water condenser for 5 h; and the progress of the reaction was monitored with TLC. After completion of the reaction, methanol was removed by distillation; the mixture was cooled to room temperature, and then added to 100 mL ice-cold water. The solid product was collected by fractional distillation and washed with excess ice cold water. The amount of product was 0.42 g. White powder; yield 76.2%; mp 192–194 °C; *R*_f_ 0.75 (*n-*hexane:EtOAc, 4:1); ^1^H NMR (400 MHz, CDCl_3_): *δ* 10.44 (1H, *s*, H-9), 8.54 (1H, *s*, H-4), 7.83 (2H, *bro d*, H-5 and H-8), 7.72 (1H, *bro s*, H-7), 7.41 (1H, *bro s*, H-8), 4.75 (3H, *s*, H-10); ^13^C NMR (101 MHz, CDCl_3_): *δ* 185.3 (C-9), 157.1 (C-2), 144.9 (C-8a), 135.9 (C-4), 128.5 (C-7), 125.7 (C-5), 123.2 (C-8), 121.0 (C-6), 120.3 (C-4a), 115.9 (C-3), 49.8 (C-10).

#### Synthesis of methyl 2-chloroquinoline-3-carboxylate (16)

A 100 mL two-neck round-bottom flask was charged with methanol (6 mL), chloroquinoline-3-carbaldehyde (0.5 g, 0.00026 mmol), and sulfuric acid (2 mL); wherein the mixture was refluxed using water condenser for 2 h and progress was monitored with TLC. After finale of the reaction, methanol was detached by distillation, the mixture was allowed to cool to room temperature, and then added to 100 mL ice-cold water. The solid violet product was collected and washed with excess ice cold water. The amount of product was 0.37 g. yellow powder; yield 60.7%; mp196–198 °C; *R*_f_ 0.66 (DCM:Methanol, 99:1); ^1^H NMR (400 MHz, CDCl_3_): *δ* 7.45 (H, *s*, H-4), 7.03 (1H, *bro s*, H-8), 6.92 (1H, *bro s*, H-5), 6.77 (1H, *bro s*, H-7), 6.65 (1H, *bro s*, H-6), 3.65 (3H *s*, OMe); ^13^C NMR (101 MHz, CDCl_3_): *δ* 166.2 (C-9), 142.0 (C-2), 134.8 (C-8a), 132.3 (C-4), 126.8 (C-7), 123.3 (C-5, 8), 119.4 (C-3,C-4a), 56.05 (OMe).

### Antibacterial activity

Four pathogenic bacterial strains, two Gram negative (*E. coli* (ATCC 25922) and *Pseudomonas aeruginosa* (ATTC 27853)) and two Gram positive bacteria (*Staphylococcus aureus* (ATCC 6538) and *Streptococcus pyogenes* (ATTC 19615) were supplied by Adama Regional Microbiology Laboratory. In vitro antibacterial activity of the synthesized compounds was done using paper disc diffusion method following the procedure reported by Zeleke et al. [18].

The bacterial cultures were inoculated into the nutrient broth (inoculation medium) and incubated overnight at 37 °C. Inoculated medium containing 24 h grown culture were added aseptically to the nutrient medium and mixed thoroughly to get a uniform distribution. The solution was poured to 20 mL of sterile MHA in sterile culture plates and allowed to attain room temperature. Sterile paper disc diffusion previously soaked in a known concentration (100 and 200 μg/mL per disc) synthesized compounds and standard drugs was prepared in DMSO using nutrient agar tubes and carefully placed at the center of the labeled seeded plate. The zones of growth inhibition around the disks were measured after 24 h of incubation at 37 °C for bacteria. Samples were analyzed in triplicates and inhibition zones were measured with a ruler and compared with the positive control disk (disk containing ciprofloxacin).

### Radical scavenging activity

The radical scavenging activity of the synthesized compounds was evaluated with 1,1-diphenyl-2-picryl hydrazyl (DPPH). In the process, 4 mg/100 mL solution of DPPH in methanol was prepared. Likewise each sample was dissolved in methanol and serially diluted with the DPPH solution to furnish four different concentrations (10, 5, 2.50, 1.25 μg/mL). The mixtures were shaken and allowed to stand at 37 °C for 30 min in dark oven, and absorbance was recorded at 517 nm using double beam UV–Vis spectrophotometer. The radical scavenging activity of ascorbic acid was measured at 2, 1, 0.5 and 0.3 μg/mL. Percentage inhibition of DPPH radical was determined using the following equation:

$$ {\text{Percentage inhibition }} = \frac{{A_{0} - A_{1} }}{{A_{0} }} \times 100 $$

where A_0_ is the absorbance of control reaction and A_1_ is the absorbance in the presence of test or standard sample [18].

### In silico analysis

#### In silico molecular docking

The experimental procedure followed for the in silico molecular docking analysis of the synthesized compounds was as reported by Blessy and Sharmila [19] with slight modifications. The interactions of the synthesized compounds with the proteins (PDB ID:6F86 and PDB ID:4FM9) were studied using AutoDock Vina v.1.2.0 [20]. The structures of the compounds synthesized were drawn using ChemOffice tool (ChemDraw 16.0) assigned with proper 2D orientation. The energy of each molecule was minimized using ChemBio3D and were then used as input for AutoDock Vina, in order to carry out the docking simulation. The crystal structures of *E. coli* gyrase B (PDB ID:6F86) and human topoisomerase IIα (PDB ID:4FM9) were downloaded from protein data bank. The protein preparation was done using the reported standard protocol by removing the co-crystallized ligand, selected water molecules (except 616, 641, and 665), and cofactors; the target protein file was prepared by leaving the associated residue with protein using Auto preparation of target protein file AutoDock 4.2 (MGLTools 1.5.6). The graphical user interface program was used to set the grid box for docking simulations. The grid was set so that it surrounds the region of interest in the macromolecule. The docking algorithm provided with AutoDock Vina v.1.2.0 was used to search for the best docked conformation between ligand and protein. During the docking process, a maximum of nine conformers were considered for each ligand. The conformations with the most favorable (least) free binding energy were selected for analyzing the interactions between the target receptor and ligands by Discovery Studio Visualizer and PyMOL. The ligands are represented in different color; H-bonds and the interacting residues are represented in ball and stick model representation [18].

#### In silico pharmacokinetics (ADME) and toxicological properties

The structures of synthesized compounds were changed to their canonical simplified molecular input line entry system (SMILES) then submitted to SwissADME tool to estimate in silico pharmacokinetic parameters and other molecular properties based on the methodology reported by Daina et al. and Lipinski [21, 22]. The organ toxicities and toxicological endpoints of the isolated compounds were predicted using PreADMET and OSIRIS Properties. The results were then compared with vosaroxin used as standard clinical drug.

## Results and discussion

### Synthesis of the target compounds

**Scheme 1:**
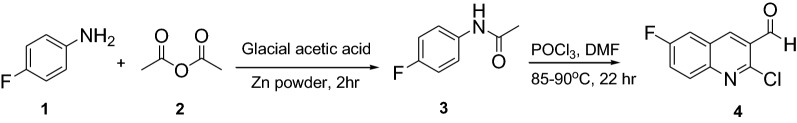
Synthesis of fluoroquinoline scaffold by Vilsmeier reaction

**Scheme 2:**
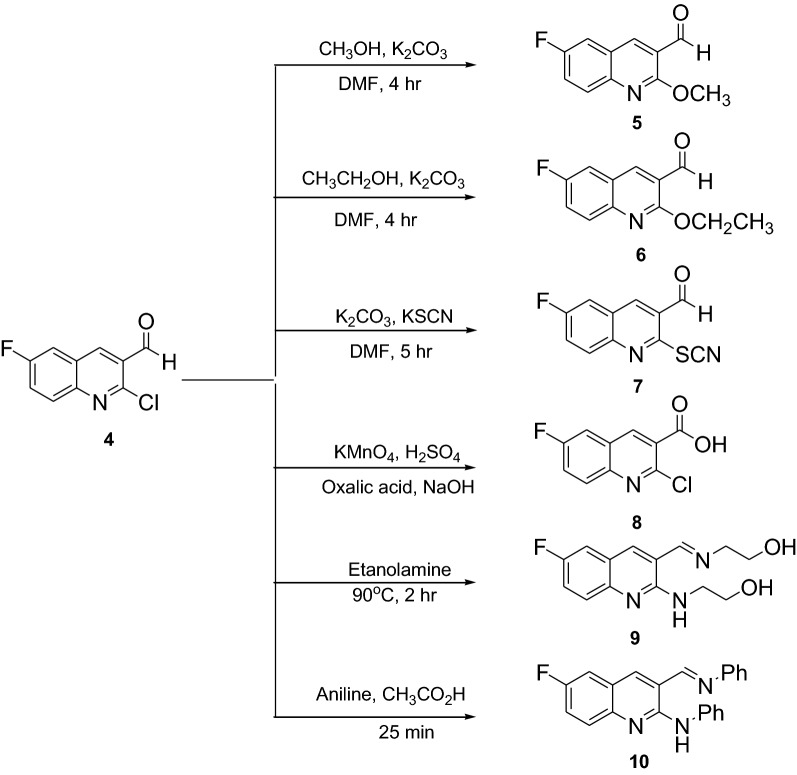
Synthesis of fluoroquinoline derivatives

**Scheme 3:**
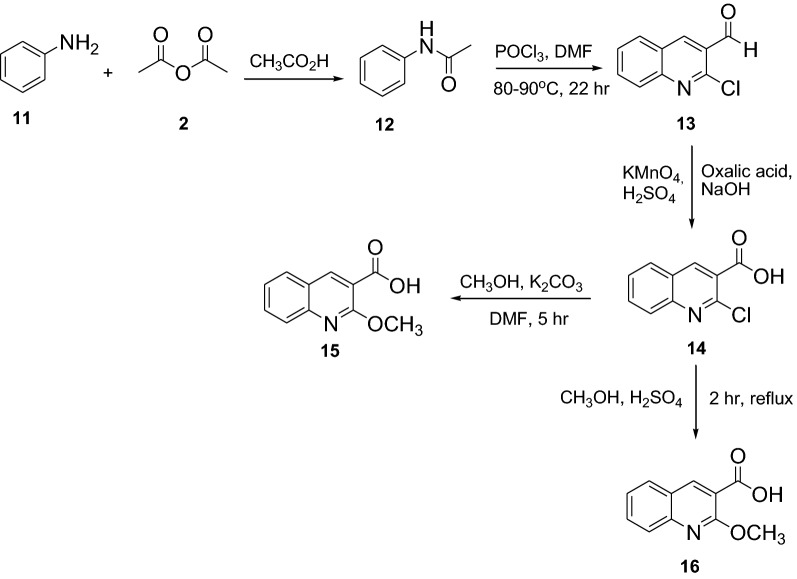
Synthesis of quinoline derivatives

The synthesis of compounds **3** and **12** started by acetylating *p*-fluoroaniline (**1**) and aniline (**11**) respectively with acetic anhydride (**2**) in the presence of glacial acetic acid and zinc powder. Vilsmeier–Haack reaction using POCl_3_ in DMF was employed for the transformation of compounds **3** and **12** to the crucial intermediate 2-chloro-6-fluoroquinoline-3-carbaldehyde (**4**) and 2-chloroquinoline-3-carbaldehyde (**13**), respectively. The chlorine atoms at position 2 of the quinolines were substituted with various nucleophiles by refluxing 2-chloro-6-fluoroquinoline-3-carbaldehyde and 2-chloroquinoline-3-carbaldehyde in *N*,*N-*dimethylformamide in basic medium. The aldehyde functional group was then oxidized to carboxylic acid (**8**) by using potassium permanganate. The chlorine in compound **8** was replaced with methoxy group using methanol in potassium carbonate to provide compound **15** while methylation using methanol in sulphuric acid gave compound **16** in good yields. Refluxing compound **4** with ethanol amine and aniline afforded imines **9** and **10**, respectively. The synthetic route employed for the preparation of a set of novel fluoroquinolines is shown in Scheme 1, 2 and 3. The structural elucidation of the synthesized compounds was accomplished using spectroscopic methods (Additional file 1).

### Antibacterial activity

Many literature reports showed that quinoline had good antimicrobial activities against various microbial pathogens [23]. In this work, an attempt was made to evaluate the compounds synthesized for their in vitro antibacterial activities against four bacterial pathogens including *S. aureus*,* E. coli*,* S. pyogene* and *P. aeruginosa* using paper disk diffusion method. Results demonstrated that the mean inhibition zone of the synthesized compounds varied from 6.34 to 15.3 mm. The mean inhibition zones displayed by the synthesized compounds were different against all of the four bacterial strains at 200 μg/mL (Table 1). Compounds **4**, **7**,** 8**, and **9** showed mean inhibition zones of 11.9 ± 0.41, 12.2 ± 0.32, 12.4 ± 0.11 and 11.2 ± 0.13 mm at 200 µg/mL against *E. coli* (Table 1). Compound **9** showed activities against three of the bacterial strains except *S. aureus.* Compounds **4**,** 5**,** 7** and **8** exhibited good activity against *S. aureus* with IZ of 12.0 ± 0.01, 10.4 ± 0.60, 11.0 ± 0.56 and 13.6 ± 0.22 mm at 200 µg/mL, respectively. On the other hand, compounds **4**,** 5**,** 6**,** 7** and **8** displayed inhibition zone of 13.7 ± 0.98, 11.8 ± 0.71 10.5 ± 0.79, 11.9 ± 0.41 and 10.4 ± 0.6 6 mm respectively against *P. aeruginosa* and compound **8** had 13.6 ± 0.22 mm against *S. aureus*. At the same concentration, the inhibition zone displayed by compound **8** against *P. aeruginosa* is better than the standard drug (ciprofloxacin) which had an IZ of 10.0 ± 0.45 mm. It was found out that fluoroquinolines showed higher bacterial inhibition zone compared with quinoline derivatives that have no fluorine substituents. The results of the present study also demonstrated that quinolines with carboxyl functional group at position-3 turned out to be more active than those derivatives containing aldehyde group at position-3.

**Table 1:** Inhibition zone of synthesized compounds in mm (mean ± SD)

Conc. μg/L	Compounds									Ciprofloxacin	Bacterial strains
	**4**	**5**	**6**	**7**	**8**	**9**	**10**	**15**	**16**		
100	11.01 ± 0.72	9.33 ± 0.33	10.50 ± 0.55	11.61 ± 0.25	11.50 ± 0.21	11.21 ± 0.11	11.01 ± 0.05	NA	NA	20.00 ± 0.76	*E. coli*
200	11.91 ± 0.41	10.01 ± 0.11	11.09 ± 0.57	12.21 ± 0.32	12.40 ± 0.11	11.32 ± 0.13	11.12 ± 0.07	7.01 ± 0.80	7.50 ± 0.65	22.22 ± 0.06	
100	12.20 ± 0.54	11.10 ± 0.32	9.76 ± 0.44	11.00 ± 0.72	11.70 ± 0.45	9.57 ± 0.75	8.90 ± 0.05	6.34 ± 0.01	6.54 ± 0.05	9.04 ± 0.14	*P. aeruginosa*
200	13.70 ± 0.98	11.80 ± 0.71	10.50 ± 0.79	11.91 ± 0.41	12.61 ± 0.52	10.40 ± 0.66	9.02 ± 0.02	7.91 ± 0.45	8.01 ± 0.55	10.05 ± 0.45	
100	11.21 ± 0.71	9.01 ± 0.64	9.31 ± 0.60	10.71 ± 0.67	13.01 ± 0.23	NA	8.88 ± 0.54	7.01 ± 0.22	7.12 ± 0.22	11.85 ± 0.91	*S. aureus*
200	12.01 ± 0.01	10.40 ± 0.60	9.70 ± 0.52	11.02 ± 0.56	13.60 ± 0.22	NA	9.30 ± 0.45	7.50 ± 0.81	7.81 ± 0.66	12.36 ± 0.53	
100	13.80 ± 0.04	12.50 ± 0.43	12.90 ± 0.35	12.30 ± 0.55	14.00 ± 0.55	9.77 ± 0.33	6.61 ± 0.87	7.11 ± 0.33	7.33 ± 0.77	17.05 ± 0.67	*S. pyogene*
200	14.70 ± 0.04	13.00 ± 0.53	13.21 ± 0.3	12.91 ± 0.33	15.33 ± 0.33	10.21 ± 0.92	7.30 ± 0.67	7.61 ± 0.99	7.90 ± 0.11	18.20 ± 0.34	

Results are mean ± SD of triplicates. Ciprofloxacin was used as positive control

**Fig. 1:**
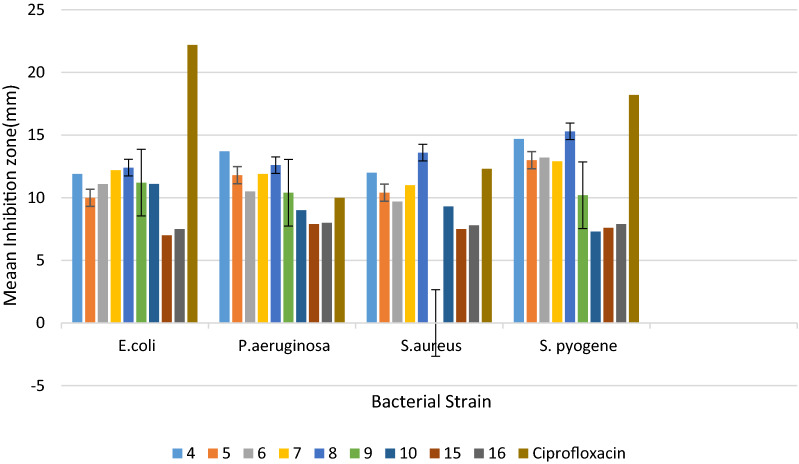
Inhibition zone (mm) of synthesized compounds at 200 µg/mL

The results of the antibacterial activity of the synthesized compounds were further presented in Fig. 1.

### In silico molecular docking analysis

#### Molecular docking studies of synthesized compounds against *E. coli* DNA gyrase B

**Fig. 2:**
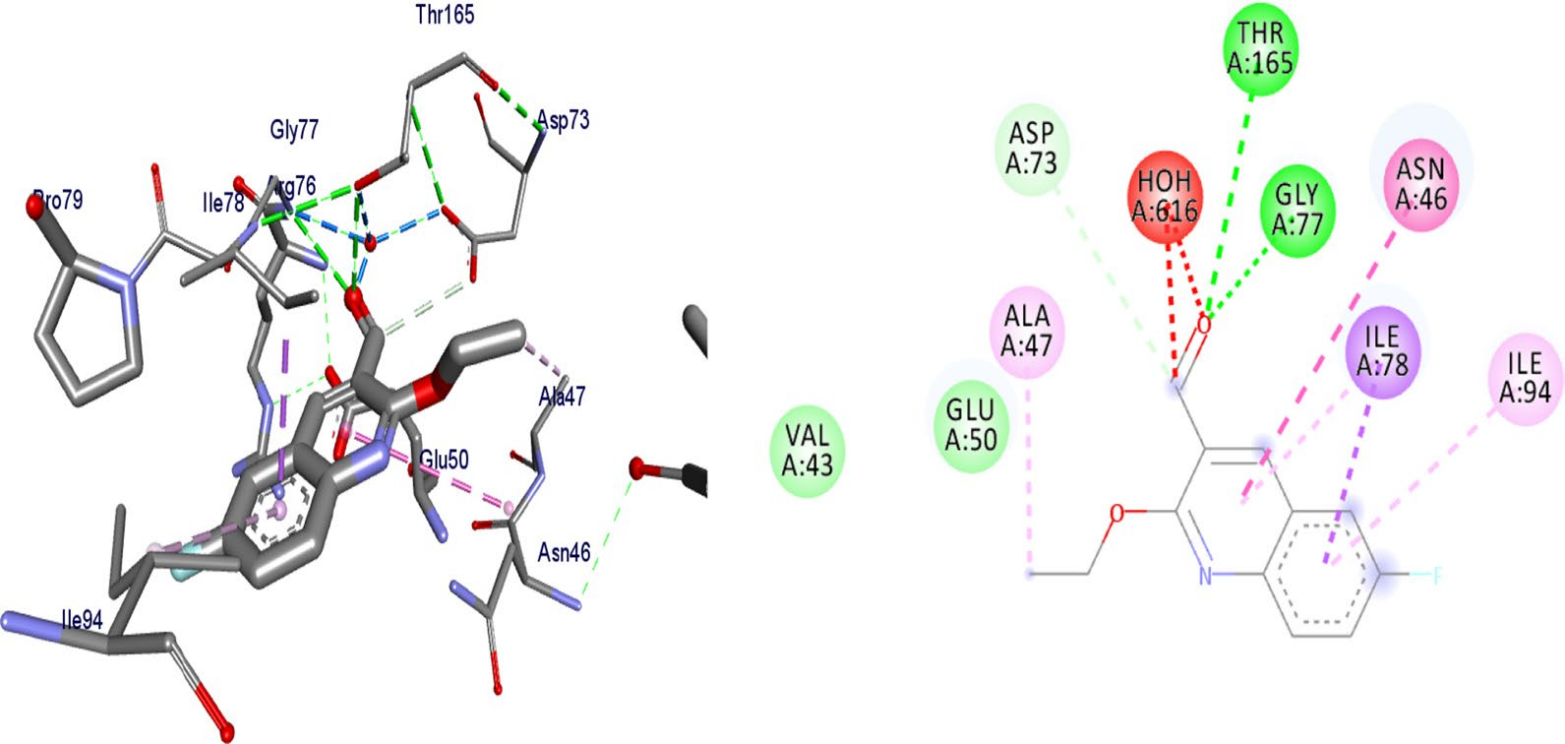
The binding interactions compound **6** against DNA gyrase B (PDB ID: 4f86)

**Fig. 3:**
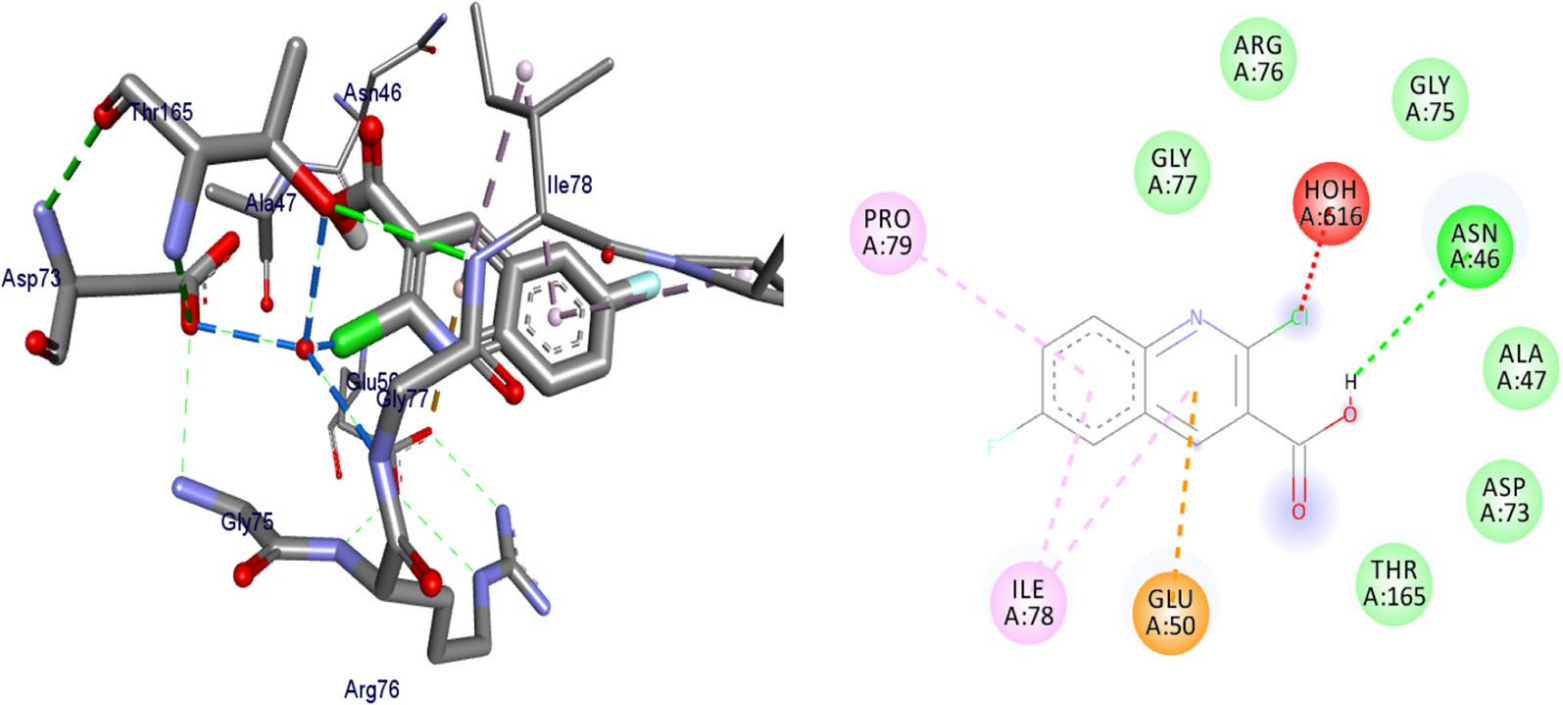
The binding interactions of compound **8** against DNA gyrase B (PDB ID: 6F86)

**Fig. 4:**
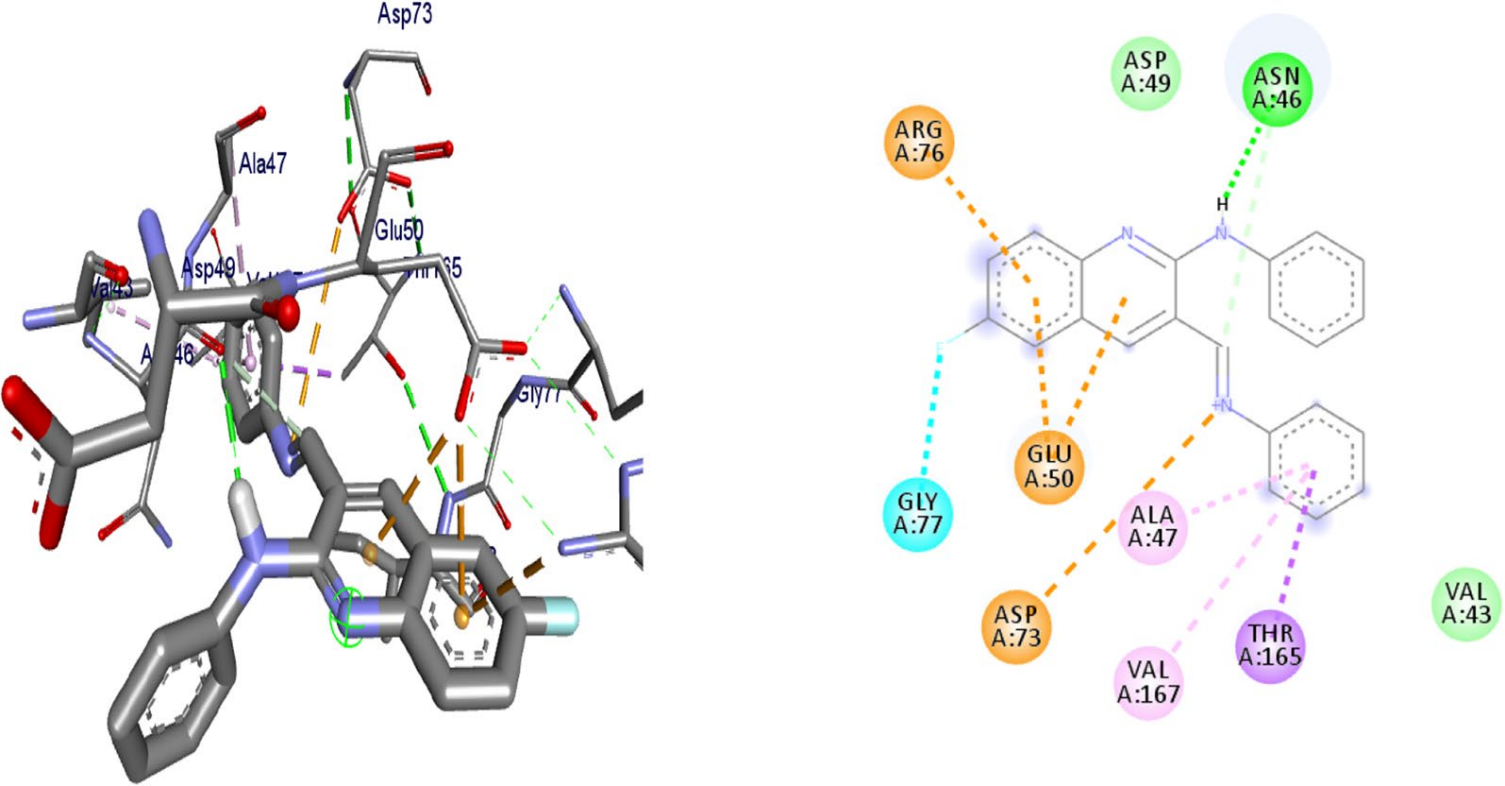
The binding interactions compound **10** against DNA gyrase B (PDB ID: 4f86)

**Fig. 5:**
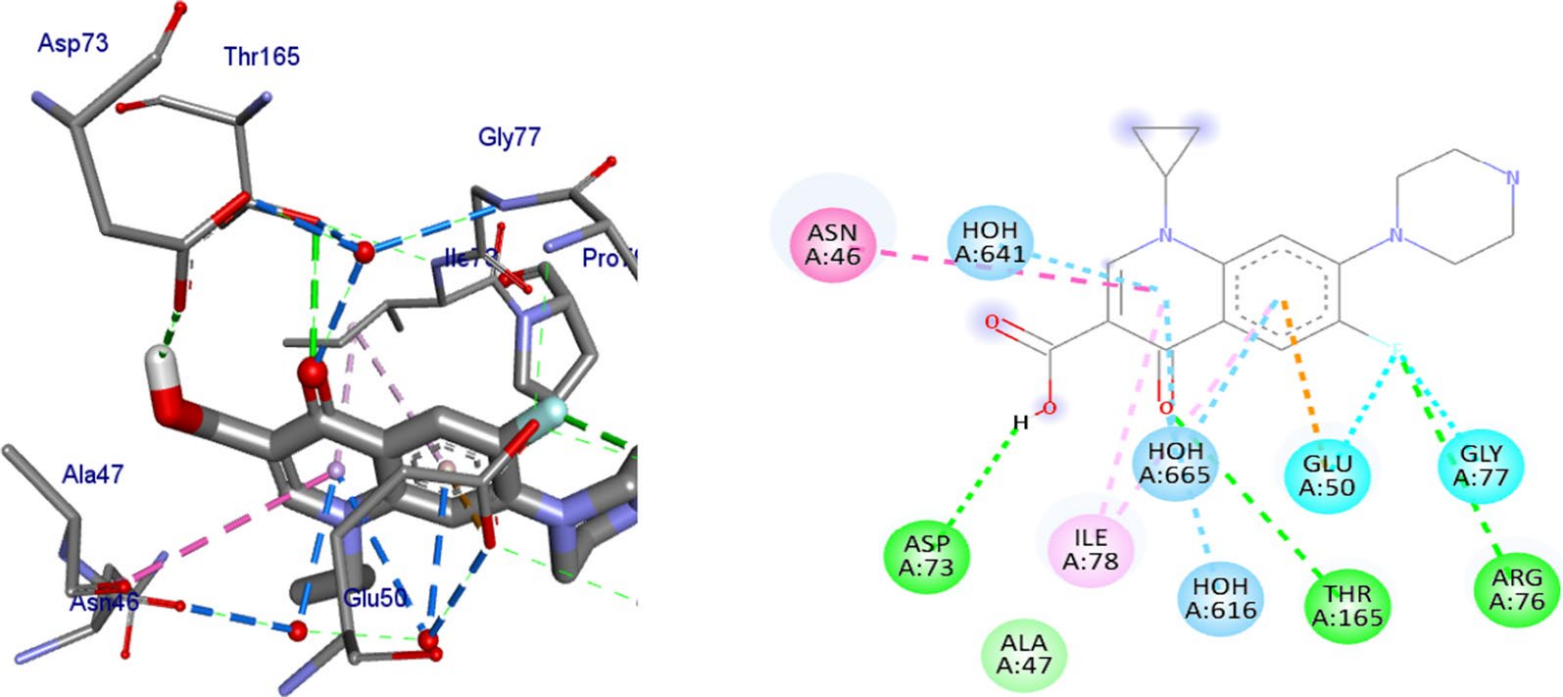
The binding interactions ciprofloxacin against DNA gyrase B (PDB ID: 4f86)

Docking is a good approach to perform in silico screening on large library of compounds and propose structural hypotheses of how the ligands inhibit the target receptors. This procedure is invaluable in lead optimization. DNA gyrase is an essential enzyme that reduces topological strain in an ATP dependent manner while double-stranded DNA is being unwound by elongating RNA-polymerase or by helicase and introduces negative supercoils into DNA [24]. The molecular docking analysis of the synthesized compounds was carried out to evaluate their binding pattern against *E. coli* DNA gyrase B and compared with standard clinical drug (ciprofloxacin) (Figs. 2, 3, 4 and 5). The binding affinities of the synthesized compounds along with their hydrogen bonding and amino acid interactions against *E. coli* DNA gyrase B were summarized in Table 2. The compounds synthesized in the present study displayed good binding affinity ranging from − 6.0 to − 7.2 kcal/mol (Table 2). Compound **10** exhibited a binding affinity with a minimum energy of − 7.2 kcal/mol which was found to be similar with the binding affinity exhibited by ciprofloxacin; the standard drug. Compared ciprofloxacin the synthesized compounds showed similar residual interactions with amino acid residues Ile-78, Asn-46 Glu-50, Asp-73 and Gly-77 and H-bond Asp-73, Arg-76 and Thr-165. Compounds **4**, **8**, **10** and **16** have additional hydrogen bonding interaction with amino acid residue Asn-46. The compounds **7** (Asp-73, Asn-46, Gly-77), **8** (Asn-46), **9 **(Asp-73, Asn-46, Glu-50, Thr-165), **10** (Asn-46) and **16** (Asp-73, Asn-46, Thr-165) exhibited additional hydrogen bonding interaction with amino acid residue. The compounds **4** (Asn-46, Ile-94, Ile-78, Ala-47),** 5** (Asn-46, Ile-94, Ile-78, Ala-47),** 6** (Asn-46, Ile-94, Ile-78, Ala-47) and **15** (Asn-46, Ile-94, Ile-78, Ala-47) have additional hydrophobic interaction with amino acid residue. The residual amino acid interactions of synthesized compounds with DNA gyrase (6f86) in this study were well in agreement with binding modes that include crucial interaction between the ligand, AS-73 and water molecules. Furthermore, compounds **8** (− 6.2 kcal/mol), **5** (− 6.3 kcal/mol), **6** (− 6.5 kcal/mol), **7** (− 6.4 kcal/mol), **9** (− 6.3 kcal/mol) and **10** (− 7.2 kcal/mol) showed comparable binding affinity with ciprofloxacin against *E. coli* DNA gyrase B. Therefore these compounds potentially be used a candidate for further study as antibacterial agents.

**Table 2:** Molecular docking results of compounds synthesized against *E. coli* DNA gyrase B (PDB ID:6F86)

Compounds	Affinity (kcal/mol)	H-bonds	Amino acid interactions	
			Hydrophobic/Pi-cation/Pi-anion/ Pi-alkyl interactions	Van der Waals interactions
**4**	− 6.1	Asp-73, Gly-77, Thr-165	Asn-46, Ile-94, Ile-78, Ala-47	Arg-76, Pro-79
**5**	− 6.3	Asp-73, Gly-77, Thr-165	Asn-46, Ile-94, Ile-78, Ala-47	Glu-50, Ala-47, Arg-76, Pro-79
**6**	− 6.5	Asp-73, Gly-77, Thr-165	Asn-46, Ile-94, Ile-78, Ala-47	Val-43, Glu-50
**7**	− 6.4	Asp-73, Asn-46, Gly-77	Ile-78, Pro-79,	Ala-47, Thr-165, Glu-50, Gly-50, Arg-76
**8**	− 6.2	Asn-46	Glu-50, Ile-78, Pro-79	Asp-73, Ala-47, Arg-76, Gly-77, Gly-75, Thr-165
**9**	− 6.3	Asp-73, Asn-46, Glu-50, Thr-165	Ile-78, Ile-94, Gly-77	Gly-75, Val-167
**10**	− 7.2	Asn-46	Ala-47, Glu-50, Asp-73, Thr-165, Gly-77, Arg-76	Asp-49, Val-43
**15**	− 6.2	Asp-73, Gly-77, Thr-165	Asn-46, Ile-94, Ile-78, Ala-47	Pro-79, Arg-76, Gly-75
**16**	− 6.0	Asp-73, Asn-46, Thr-165	Glu-50, Arg-76, Ile-78	–
**Ciprofloxacin**	− 7.2	Asp-73, Arg-76, Thr-165	Glu-50, Gly-77, Ile-78, Asn-46	Ala-47

Binding interactions of the synthesized compounds and ciprofloxacin against DNA gyrase B were shown in Figs. 2, 3, 4 and 5. Ribbon model shows the binding pocket structure of DNA gyrase B with compounds. Hydrogen bonds between compounds and amino acids are shown as green dashed lines, hydrophobic interaction are shown as pink lines.

#### Molecular docking results of synthesized compounds against human topoisomerase IIα

Topoisomerases are target for cancer therapy by selectively cleaving, rearranging, and relegating DNA strands. Topoisomerases can both alter the super helical state of DNA and help disentangle interlinked chromosomes. Topoisomerases are categorized as type I or type II [25]. In this project, molecular docking interactions of the synthesized compounds against human topoisomerase IIα were studied and compared with vosaroxin used as an anticancer drug. The synthesized compounds were found to have binding affinity ranging from − 6.8 to − 7.5 (kcal/mol) (Table 3). The best results were displayed by compound **5** (− 7.2 kcal/mol), **6** (− 7.2 kcal/mol), **8** (− 7.4 kcal/mol), **10** (− 7.4 kcal/mol) and **16** (− 7.5 kcal/mol). Compound **5** and **6** have similar binding affinity with vosaroxin (− 7.2 kcal/mol). Compound **10** and **16** have better binding affinity than vosaroxin. Compared to vosaroxin, the synthesized compounds showed similar residual interactions with amino acid residues Leu-705, Ile-577, Pro-593, Glu-682, Tyr-684, Arg-672, and Gln-542 and H-bonding interaction with Leu-705, Ile-577 and Pro-593. Compounds **6**,** 10** and **16** have additional hydrogen bonding interaction with amino acid residue Leu-705. The synthesized compounds showed similar residual interactions with vosaroxin against Human Topoisomerase IIα. The residual amino acid interactions of the synthesized compounds in this study were well in agreement with binding modes that include crucial hydrogen interaction between the ligand and amino acids residues (Ser-591 and Leu-685). Compounds **5**, **6**,** 8**,** 10** and **16** might be better anticancer agents than other synthesized compounds and need further studies.

**Table 3:** Molecular docking results of the compounds synthesized against human topoisomerase IIα (PDB ID:4fm9)

Compounds	Affinity (kcal/mol)	H-bonds	Amino acid interactions	
			Hydrophobic/Pi-cation/Pi-anion/ Pi-alkyl interactions	Van der Waals interactions
**4**	− 6.8	Ser-547	Ile-577, Gln-542, Tyr-686, Leu-685, Leu-705, Lys-701	Ser-591, Pro-593, Leu-592, Tyr-590, Gln-544, Asp-543, Glu-702
**5**	− 7.2	Ser-547, Glu-702, Gln-542, Leu-592, Tyr-684	Asp-683, Leu-685, Leu-705	Asp-543, Ile-577, Lys-701
**6**	− 7.2	Ser-547, Leu-685	Leu-705, Lys-701	Tyr-684, Glu-702, Tyr-686, Gln-542, Asp-543, Gln-544, Ile-577
**7**	− 7.1	Ser-547, Asp-543, Leu-685	Leu-705, Lys-701	Asp-683, Tyr-684, Glu-702, Gln-542, Ile-577, Gln-544, Tyr-590
**8**	− 7.4	–	Glu-586, Met-587, Ala-588, Trp-598, Val-580, Phe-589	Phe-595, Val0578, Tyr-590,
**9**	− 7.0	Ser-547, Ser-591, Asp-543, Gln-542, Pro-593, Leu-685	Asp-683, Lys-701, Leu-705, Glu-702	Gly-546, Asp-541, Ile-577, Tyr-684
**10**	− 7.4	–	Asp-541, Gln-542, Asp-543, Leu-592, Pro-593, Leu-705	Ser-547, Glu-702, Tyr-686, Asp-683, Lys-701, Leu-685, Tyr-684, Gln-544
**15**	− 6.9	Ser-547, Gln-542, Leu-685	Leu-705	Asp-543, Glu-702, Tyr-686, Lys-702, Tyr-684, Tyr-590, Ile-577
**16**	− 7.5	Ser-547, Glu-702, Asp-543, Leu-685, Gln-542, Leu-592	Ile-577, Leu-705, Pro-593	Lys-701, Tyr-684, Tyr-590
**Vosaroxin**	− 7.2	Ser-591, Leu-685, Leu-592	Leu-705, Ile-577, Pro-593	Glu-682, Tyr-684, Arg-672, Gln-542

**Fig. 6:**
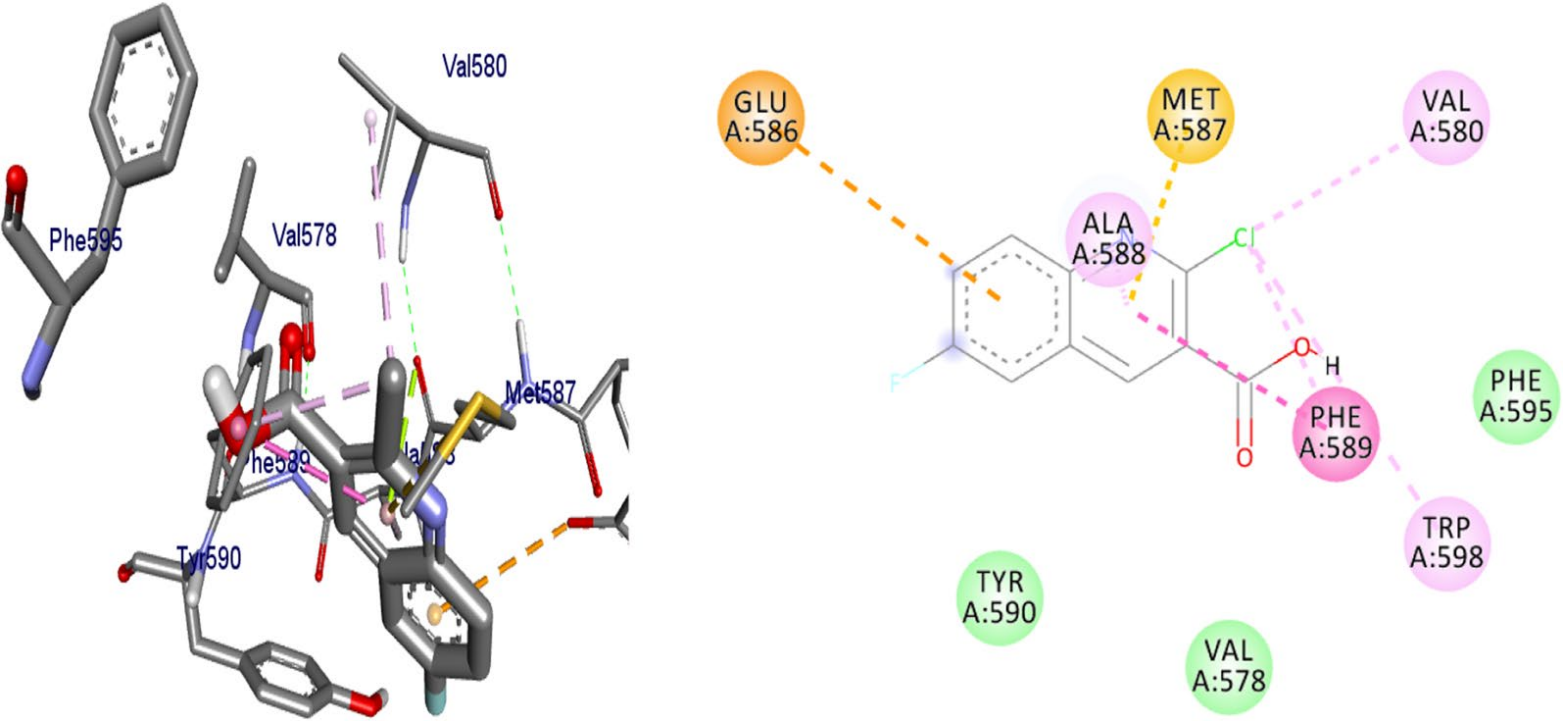
The binding interactions compound **8** against human topoisomerase IIα

**Fig. 7:**
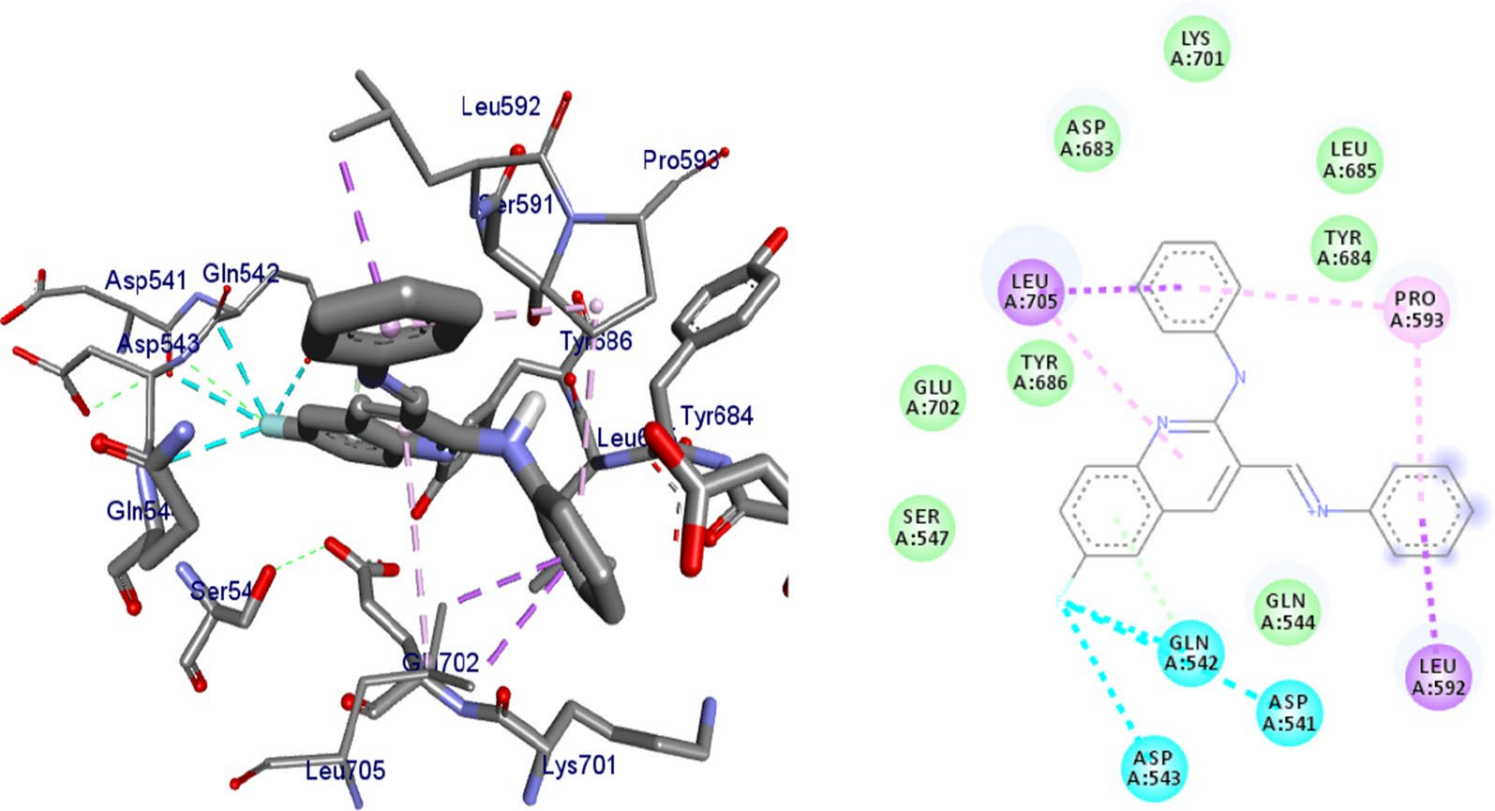
The binding interactions compound **10** against human topoisomerase IIα

**Fig. 8:**
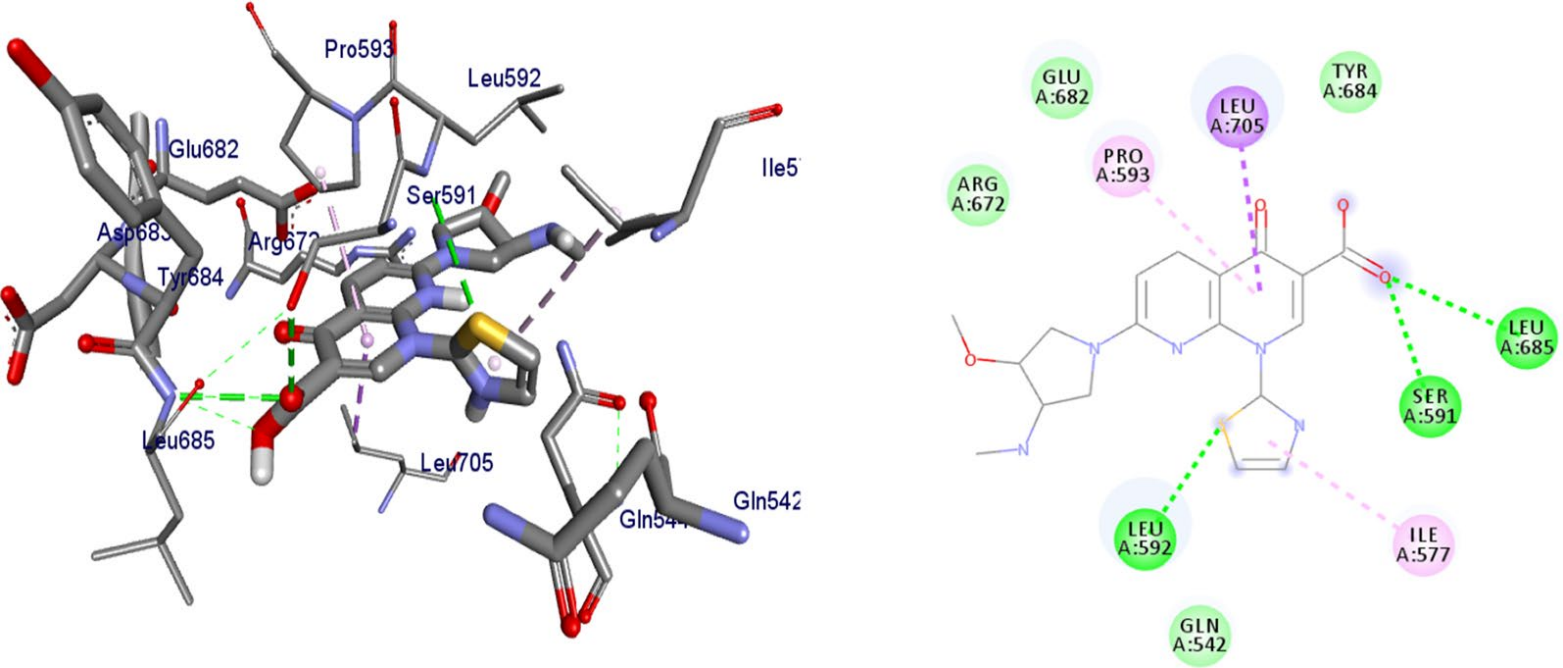
The binding interactions of vosaroxin against human topoisomerase Iiα

Binding interactions of synthesized compounds and vosaroxin against human topoisomerase IIα were shown in Figs. 6, 7 and 8. The Ribbon model shows the binding pocket structure of human topoisomerase IIα with compounds. Hydrogen bonds between compounds and amino acids are shown as green dashed lines, hydrophobic interaction are shown as pink lines.

#### Pharmacokinetic properties of synthesized compounds

The process of designing and developing a drug is not only time consuming but also it demands huge human and material resources. One of the major challenges in this regard is the evaluation of its ADMET properties in humans. Nowadays, the pharmacokinetic properties of compounds can be analyzed using computer assisted in silico screenings methods. In the course of this work, the compounds synthesized were analyzed for their drug likeness properties using SwissADME predictions. The results were analyzed using Lipinski’s rule of five. Lipinski defines these compounds as drug like; having sufficiently acceptable ADMET properties to survive through the Phase I clinical trials. The rule predicts high probability of success or failure due to drug likeness for molecules complying with 2 or more of the following rules: molecular mass less than 500 Dalton, high lipophilicity (expressed as logP less than 5), less than 5 hydrogen bond donors, less than 10 hydrogen bond acceptors and molar refractivity should be between 40–130 [26, 27] (Additional file 1).

**Fig. 9:**
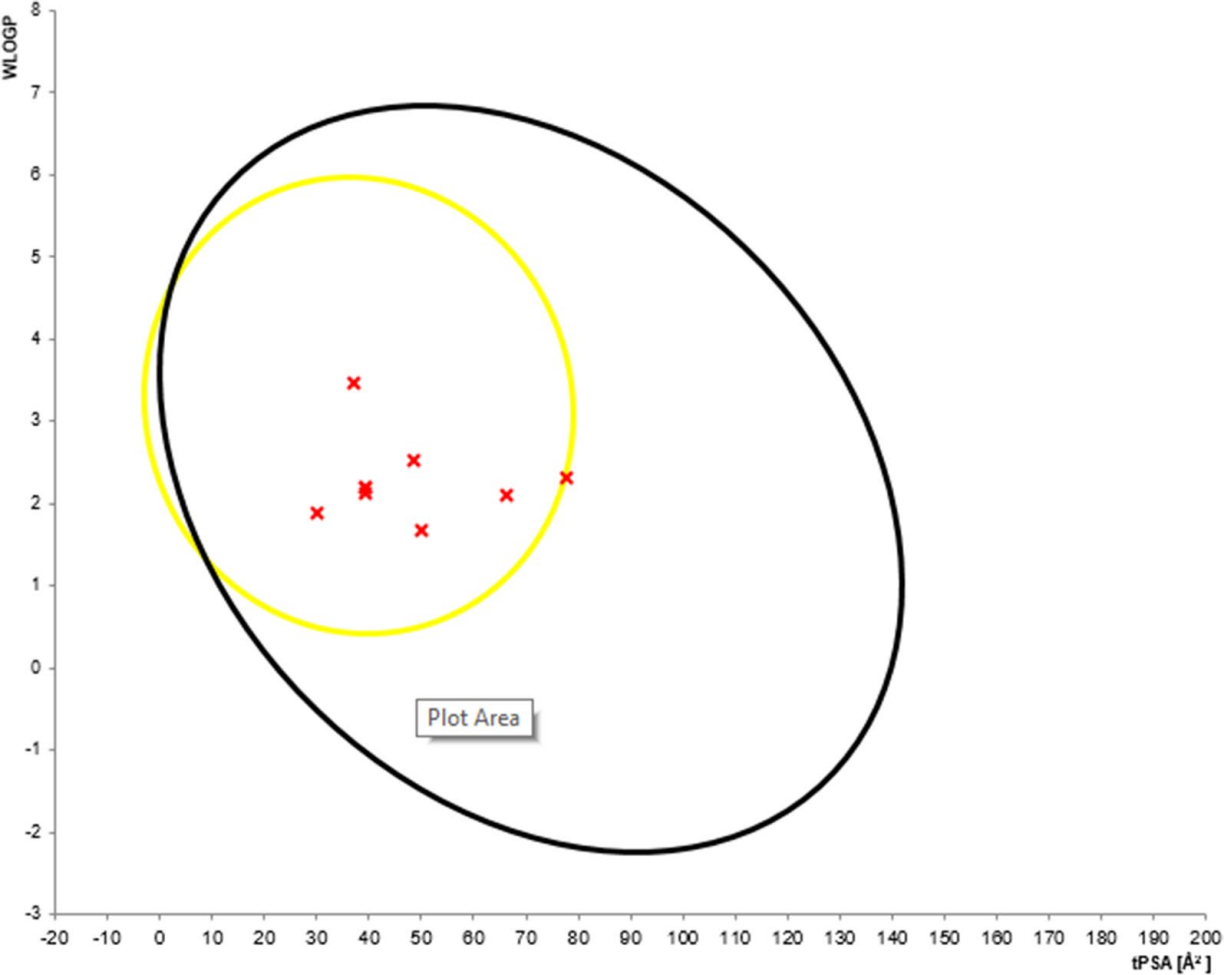
BOILED-Egg model for predicting gastrointestinal absorption and brain access

SwissADME predictions show that all synthesized compounds satisfy Lipinski’s rule of five with zero violation (Table 4). From the results obtained, clogp values of all the synthesized compounds were found to be less than 5, molecular weight of all the derivatives were less than 500 Daltons, hydrogen bond donor and acceptor is not more than 5 and 10 respectively. All of the compounds synthesized satisfy Lipinski’s rule of five. The skin permeation value (log Kp) of synthesized compounds were found to be in the range of − 4.5 to − 7.2 cm/s. This clearly shows low skin permeability of the synthesized compounds. Additionally, all the compounds were also satisfying the Veber’s rule (fewer (< 10) rotatable bonds, lesser polar surface area (< 140 A) and fewer H-bond donors and acceptors) [28]. The predicted logP values revealed that compounds have optimal liphophilicity (ranging from 1.89 to 3.47). Besides, SwissADME predictions parameters showed that all the compounds have high gastrointestinal (GI) absorption, blood brain barriers (BBB) permeation (except compound **9** and **10**). As per the reports from the Veber’s rule, fewer the rotatable bonds and lesser the TPSA correlate to higher permeation and gastrointestinal absorption [28]. The results show that compounds with fewer rotatable bond < 6 shows high GI absorption and BBB permeation. The brain or intestine estimated permeation graph prediction model (BOILED-Egg) shows the correlation between the prediction of gastrointestinal (GI) absorption and blood–brain barrier (BBB) penetration with the alignment between lipophilicity (WlogP) and polarity (TPSA) properties. The BOILED-Egg prediction model shows that except compound **6**, all other compounds were within the yolk indicating better permeation (Fig. 9). Except compound **9**, no compounds are substrate of permeability to glycoprotein. It was also found that all of the compounds have inhibitor interaction (CYP1A2 inhibitor) except compound **9**. All the compounds haven’t inhibitor interaction (CYP2C9, CYP2D6 and CYP3A4 inhibitor) (except compound **9**) (Table 5).

**Table 4:** Drug-likeness predictions of compounds computed by SwissADME

S. no	Formula	Mol. wt. (g/mol)	NRB	NHA	NHD	TPSA (A^°2^)	LogP (cLogP)	Lipinski’s rule of five violation
4	C_10_H_5_ClFNO	209.6	1	3	0	29.96	1.89	0
5	C_11_H_8_FNO_2_	205.19	2	4	0	39.19	2.20	0
6	C_12_H_10_FNO_2_	219.21	3	4	0	39.19	2.19	0
7	C_12_H_6_FNOS	231.25	2	3	0	66.16	2.10	0
8	C_10_H_5_ClFNO_2_	225.60	1	4	1	50.19	1.67	0
9	C_14_H_16_FN_3_O_2_	277.29	6	5	3	77.74	2.31	0
10	C_22_H_16_FN_3_	341.38	4	3	1	37.28	3.47	0
15	C_11_H_9_NO_2_	187.19	2	3	0	39.19	2.12	0
16	C_12_H_11_NO_3_	217.22	3	4	0	48.42	2.52	0
Vosaroxin	C_18_H_19_N_5_O_4_S	401.45	5	9	2	136.13	0.963	0

*NHD* number of hydrogen donor; *NHA* number of hydrogen acceptor

**Table 5:** ADME predictions of compounds computed by SwissADME and PreADMET

Compounds	Formula	Skin permeation value (log Kp) cm/s	GI absorption	BBB permeability	Inhibitor interaction (SwissADME/PreADMET)					
					P-gp substrate	CYP1A2	CYP2C19	CYP2C9	CYP2D6	CYP3A4
**4**	C_10_H_5_ClFNO	− 5.65	High	Yes	No	Yes	No	No	No	No
**5**	C_11_H_8_FNO_2_	− 6.09	High	Yes	No	Yes	No	No	No	No
**6**	C_12_H_10_FNO_2_	− 5.92	High	Yes	No	Yes	Yes	No	No	No
**7**	C_12_H_6_FNOS	− 5.37	High	Yes	No	Yes	Yes	No	No	No
**8**	C_10_H_5_ClFNO_2_	− 5.7	High	Yes	No	No	No	No	No	No
**9**	C_14_H_16_FN_3_O_2_	− 7.42	High	No	Yes	No	No	No	No	No
**10**	C_22_H_16_FN_3_	− 4.5	High	No	No	Yes	Yes	No	Yes	Yes
**15**	C_11_H_9_NO_2_	− 6.05	High	Yes	No	Yes	No	No	No	No
**16**	C_12_H_11_NO_3_	− 5.96	High	Yes	No	Yes	No	No	No	No
**Vosaroxin**	C_18_H_19_N_5_O_4_S	− 8.98	High	No	Yes	Yes	No	No	No	No

*NRB* number of rotatable bonds; *TPSA* total polar suRface area

All of the synthesized compounds were computed by Pro-Tox II and OSIRIS property to evaluate their toxicity like hepatotoxicity, carcinogenicity, immunotoxicity, mutagenicity, and cytotoxicity. Toxicological prediction results suggested that all compounds are more or less non-hepatotoxic, non-carcinogenic, immunogenic and non-cytotoxic suggesting these compounds as lead for further study (Table 6).

**Table 6:** Toxicity prediction of compounds computed by Pro-Tox II and OSIRIS property explorer

S. no	Formula	LD_50_ (mg/kg)	Toxicity class	Organ toxicity				
				Hepatotoxicity	Carcinogenicity	Immunotoxicity	Mutagenicity	Cytotoxicity
**4**	C_10_H_5_ClFNO	2190	5	Inactive	Inactive	Inactive	Inactive	Inactive
**5**	C_11_H_8_FNO_2_	1000	4	Active	Active	Active	Inactive	Inactive
**6**	C_12_H_10_FNO_2_	800	4	Inactive	Active	Inactive	Inactive	Inactive
**7**	C_12_H_6_FNOS	80	3	Active	Inactive	Inactive	Inactive	Inactive
**8**	C_10_H_5_ClFNO_2_	2190	5	Active	Inactive	Inactive	Inactive	Inactive
**9**	C_14_H_16_FN_3_O_2_	756	4	Inactive	Inactive	Active	Inactive	Active
**10**	C_22_H_16_FN_3_	250	3	Active	Inactive	Inactive	Active	Inactive
**15**	C_11_H_9_NO_2_	1000	4	Inactive	Active	Inactive	Inactive	Inactive
**16**	C_12_H_11_NO_3_	800	4	Inactive	Active	Inactive	Inactive	Inactive
**Vosaroxin**	C_18_H_19_N_5_O_4_S	500	4	Active	Inactive	Inactive	Inactive	Inactive

### Radical scavenging activity of the synthesized compounds

Compounds **4**, **5**, **6**, **7**, **8**, **9** and **10** exhibited percentage inhibition that is comparable to ascorbic acid suggesting their strong antioxidant activity. Among them, compound **7** and **8** had strongest antioxidant activity with IC_50_ values of 5.31 and 5.41 μg/mL, respectively (Table 7). The high radical scavenging activity of these compounds might be due to the presence of thiocyanate in compound **7** and carboxyl functional group in compound **8**. Likewise, the percentage radical scavenging activity of ascorbic acid was found to be 80.8 at 10 μg/mL. The results obtained are comparable with ascorbic acid with IC_50_ value of 1. This indicates that the synthesized compounds are strong radical scavengers.

**Table 7:** Radical scavenging activity and IC_50_ values of the synthesized compounds and ascorbic acid

Con. μg/mL	Compounds									
	**4**	**5**	**6**	**7**	**8**	**9**	**10**	**15**	**16**	**Ascorbic acid**
1.25	35.86	30.45	47.15	48.01	47.52	37.67	43.21	20.17	17.30	40.2
2.5	47.70	42.54	55.95	56.51	56.01	45.13	55.24	23.75	23.22	51.5
5	51.56	50.42	62.41	63.11	62.67	55.53	60.22	26.60	26.81	74.4
10	62.46	61.43	71.12	73.32	72.02	57.78	66.09	35.55	35.12	80.8
IC_50_	6.35	6.65	5.48	5.31	5.41	6.54	5.75	16.71	11.09	2.07

**Fig. 10:**
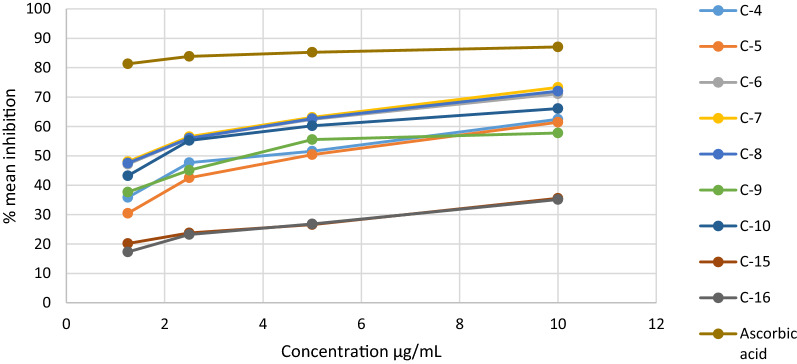
The percentage inhibition of the compounds and ascorbic acid

The percentage radical scavenging activity and IC_50_ values of the synthesized compounds were further displayed in Fig. 10.

## Conclusion

In conclusion, we have synthesized nine novel fluoroquinolines and chloroquinoline derivatives with their structures established using spectroscopic methods. Compounds **4**–**9** exhibited activity against both Gram positive and Gram negative bacterial strains indicating the action of these compounds over a wide range of disease-causing bacteria. The activity displayed by these compounds might be due to the presence of fluorine in compounds **4**–**9**. Compounds **5**–**10** showed comparable binding affinities in their in silico molecular docking analysis against *E. coli* DNA gyrase B. They also satisfy Lipinski’s rule of five without violation which suggests that these compounds could possibly be antibacterial agents; to be verified by further study. Compounds **7** and **8** were proved to be very potent radical scavenger with IC_50_ values of 5.31 and 5.41 μg/mL; respectively, with their activities comparable with the activities of ascorbic acid used as positive control. Compound **5**, **6**,** 8**,** 10** and **16** had comparable binding affinity with vosaroxin against human topoisomerase IIα suggesting the compounds as a possible candidate for anticancer drugs.

## Supplementary Information

**Additional file 1**. Additional Appendix S1–S7.

## Data Availability

All the data obtained and materials investigated in this research are accessible with the corresponding author.

## Abbreviations

ADMET: Absorption, distribution, metabolism, excretion, and toxicity
DNA: Deoxyribonucleic acid
DPPH: 1, 1-Diphenyl-2-picryl hydrazyl
HBA: H-bond acceptors
HBD: H-bond donors
IC_50_: Inhibitory concentration inhibiting 50% of growth
NMR: Nuclear magnetic resonance
TLC: Thin layer chromatography
VR: Vilsmeier reagents
