# Supplementary material for: Synthesis, in silico molecular docking analysis, pharmacokinetic properties and evaluation of antibacterial and antioxidant activities of fluoroquinolines
Source: BMC Chem. 2022 Jan 13;16(1):1. doi: 10.1186/s13065-022-00795-0 (PMC8759279; doi:10.1186/s13065-022-00795-0)
Supplement: Supplementary file 1 — Additional file 1. Additional Appendix S1–S7. [file 13065_2022_795_MOESM1_ESM.docx]

**Additional file**

Herein we have presented NMR spectra of the synthesized compounds as supplementary information

**Appendix S1:** ^1^H NMR spectrum (400 MHz, CDCl_3_ and CD_3_OD) of 2-chloro-6-fluoroquinoline-3-carbaldehyde (**4**)

**Appendix S2:** ^13^C NMR spectrum (100 MHz, CDCl_3_) of 2-chloro-6-fluoroquinoline-3-carbaldehyde (**4**)

**Appendix S4:** ^1^H NMR spectrum (400 MHz, CDCl_3_) of 6-fluoro-2-methoxyquinoline-3-carbaldehyde (**5**)

**Appendix S5:** ^13^C NMR spectrum (100 MHz, CDCl_3_) of 6-fluoro-2-methoxyquinoline-3-carbaldehyde (**5**)

**Appendix S6:** DEPT-135 NMR spectrum of 6-fluoro-2-methoxyquinoline-3-carbaldehyde (**5**)

**Appendix S7**: ^1^H NMR spectrum (400 MHz, CDCl_3_) of 2-ethoxy-6-fluoroquinoline-3-carbaldehyde (**6**)

**Appendix S8**: ^13^C NMR spectrum (100 MHz, CDCl_3_) of 2-ethoxy-6-fluoroquinoline-3-carbaldehyde (**6**)

**Appendix S9:** DEPT-135 NMR spectrum of 2-ethoxy-6-fluoroquinoline-3-carbaldehyde (**6**)

**Appendix S10**:  ^1^H NMR spectrum (400 MHz, CDCl_3_) of 6-fluoro-2-thiocyanatoquinoline-3-carbaldehyde (**7**)

**Appendix S11:** ^13^C NMR spectrum (100 MHz, CDCl_3_) of 6-fluoro-2-thiocyanatoquinoline-3-carbaldehyde (**7**)

**Appendix S12:** DEPT-135 NMR spectrum of 6-fluoro-2-thiocyanatoquinoline-3-carbaldehyde (**7**)

**Appendix S13:** ^1^H NMR spectrum (400 MHz, CDCl_3_) of 2-chloro-6-fluoroquinoline-3-carboxylic acid (**8)**

**Appendix S14**:^13^C-NMR spectrum (100 MHz, CDCl_3_) of 2-chloro-6-fluoroquinoline-3-carboxylic acid (**8**)

**Appendix S15:** DEPT-135 spectrum of 2-chloro-6-fluoroquinoline-3-carboxylic acid (**8**)

**Appendix S16**: ^1^H NMR spectrum (400 MHz, CDCl_3_) of 2-((2-hydroxyethyl) amino)-3-(-2-(ethylideneamino) ethanol quinoline **(9**)

**Appendix S17:** ^13^C NMR spectrum (100 MHz, CDCl_3_) of 2-((2-hydroxyethyl) amino)-3-(-2-(ethylideneamino) ethanol quinoline (**9**)

**Appendix S18:** DEPT-135 NMR spectrum of 2-((2-hydroxyethyl) amino)-3-(-2-(ethylideneamino) ethanol quinoline (**9**)

**

**Appendix S19:** ^1^H NMR spectrum (400 MHz, CDCl_3_) of 6-fluoro-N-phenyl-3-((phenylimino) methyl) quinolin-2-amine (**10**)

**Appendix S20:** ^13^C NMR spectrum (100 MHz, CDCl_3_) of 6-fluoro-N-phenyl-3-((phenylimino) methyl) quinolin-2-amine (**10**)

**Appendix S22:** ^1^H NMR spectrum (400 MHz, CDCl_3_) of 2-methoxyquinoline-3-carboxylic acid (**15**)

**Appendix S23**: ^13^C NMR spectrum (100 MHz, CDCl_3_) of 2-methoxyquinoline-3-carboxylic acid (**15**)

**Appendix S24:** DEPT-135 spectrum of 2-methoxyquinoline-3-carboxylic acid (**15**)

**Appendix S26:** ^13^C NMR spectrum (100 MHz, CDCl_3_) of methyl 2-chloroquinoline-3-carboxylate (**16**)

**Appendix S27:** DEPT-135 spectrum of methyl 2-chloroquinoline-3-carboxylate (**16**)
